# Reducing the risk of *Plasmodium vivax* after falciparum infections in co-endemic areas—a randomized controlled trial (PRIMA)

**DOI:** 10.1186/s13063-022-06364-z

**Published:** 2022-05-18

**Authors:** Kamala Thriemer, Tamiru Shibru Degaga, Michael Christian, Mohammad Shafiul Alam, Benedikt Ley, Mohammad Sharif Hossain, Mohammad Golam Kibria, Tedla Teferi Tego, Dagimawie Tadesse Abate, Sophie Weston, Amalia Karahalios, Megha Rajasekhar, Julie A. Simpson, Angela Rumaseb, Hellen Mnjala, Grant Lee, Rodas Temesgen Anose, Fitsum Getahun Kidane, Adugna Woyessa, Kevin Baird, Inge Sutanto, Asrat Hailu, Ric N. Price

**Affiliations:** 1 Global and Tropical Health Division, Menzies School of Health Research and Charles Darwin University, Darwin, Australia; 2 College of Medicine & Health Sciences, Arba Minch University, Arba Minch, Ethiopia; 3 Eijkman-Oxford Clinical Research Unit, Jakarta, Indonesia; 4 International Centre for Diarrhoeal Disease Research, Dhaka, Bangladesh; 5 Arba Minch General Hospital, Arba Minch, Ethiopia; 6 Centre for Epidemiology and Biostatistics, Melbourne School of Population and Global Health, The University of Melbourne, Melbourne, Victoria Australia; 7 Ethiopian Public Health Institute, Addis Ababa, Ethiopia; 8 Centre for Tropical Medicine and Global Health, Nuffield Department of Clinical Medicine, University of Oxford, Oxford, UK; 9 Department of Parasitology, Faculty of Medicine, University of Indonesia, Jakarta, Indonesia; 10 College of Health Sciences, Addis Ababa University, Addis Ababa, Ethiopia; 11 Mahidol-Oxford Tropical Medicine Research Unit, Faculty of Tropical Medicine, Mahidol University, Bangkok, Thailand

**Keywords:** Vivax malaria, Falciparum malaria, Radical cure, Universal radical cure, Vivax elimination, Co-endemic, Randomized controlled trial

## Abstract

**Background:**

*Plasmodium vivax* forms dormant liver stages that can reactivate weeks or months following an acute infection. Recurrent infections are often associated with a febrile illness and can cause a cumulative risk of severe anaemia, direct and indirect mortality, and onward transmission of the parasite. There is an increased risk of *P. vivax* parasitaemia following falciparum malaria suggesting a rationale for universal use of radically curative treatment in patients with *P. falciparum* malaria even in the absence of detectable *P. vivax* parasitaemia in areas that are co-endemic for both species.

**Methods:**

This is a multicentre, health care facility-based, randomized, controlled, open-label trial in Bangladesh, Indonesia and Ethiopia. Patients with uncomplicated falciparum malaria, G6PD activity of ≥70% of the adjusted male median (AMM) and haemoglobin levels ≥8g/dl are recruited into the study and randomized to either receive standard schizonticidal treatment plus 7-day high dose primaquine (total dose 7mg/kg) or standard care in a 1:1 ratio. Patients are followed up weekly until day 63. The primary endpoint is the incidence risk of any *P. vivax* parasitemia on day 63. Secondary endpoints include incidence risk on day 63 of symptomatic *P. vivax* malaria and the risk of any *P. falciparum* parasitaemia. Secondary safety outcomes include the proportion of adverse events and serious adverse events, the incidence risk of severe anaemia (Hb<5g/dl and <7g/dl) and/or the risk for blood transfusion, the incidence risk of ≥ 25% fall in haemoglobin with and without haemoglobinuria, and the incidence risk of ≥ 25% fall in haemoglobin to under 7g/dl with and without haemoglobinuria.

**Discussion:**

This study evaluates the potential benefit of a universal radical cure for both *P. vivax* and *P. falciparum* in different endemic locations. If found safe and effective universal radical cure could represent a cost-effective approach to clear otherwise unrecognised *P. vivax* infections and hence accelerate *P. vivax* elimination.

**Trial registration:**

NCT03916003. Registered on 12 April 2019.

**Supplementary Information:**

The online version contains supplementary material available at 10.1186/s13063-022-06364-z.

## Administrative information

Note: the numbers in curly brackets in this protocol refer to SPIRIT checklist item numbers. The order of the items has been modified to group similar items (see http://www.equator-network.org/reporting-guidelines/spirit-2013-statement-defining-standard-protocol-items-for-clinical-trials/).

**Table:** 

**Title {1}**	Reducing the risk of *Plasmodium vivax* after falciparum infection in co-endemic areas - a randomized controlled trial
**Trial registration {2a and 2b}**	NCT03916003 registered 12^th^ April 2019
**Protocol version {3}**	V6.0 April 2022
**Funding {4**}	Australian Academy of Science Regional Collaborations Program Bill & Melinda Gates Foundation (OPP1164105/INV-010504) National Health and Medical Research Council (NHMRC) (GNT1132975)
**Author details {5a}**	1 Global and Tropical Health Division, Menzies School of Health Research and Charles Darwin University, Darwin Australia 2 College of Medicine & Health Sciences, Arba Minch University, Arba Minch, Ethiopia 3 Eijkman-Oxford Clinical Research Unit, Jakarta, Indonesia 4 International Centre for Diarrhoeal Disease Research, Dhaka, Bangladesh 5 Arba Minch General Hospital, Arba Minch, Ethiopia 6 Centre for Epidemiology and Biostatistics, Melbourne School of Population and Global Health, The University of Melbourne, Melbourne, Victoria, Australia 7 Ethiopian Public Health Institute, Addis Ababa, Ethiopia 8 Centre for Tropical Medicine and Global Health, Nuffield Department of Clinical Medicine, University of Oxford, Oxford UK 9 Department of Parasitology, Faculty of Medicine, University of Indonesia, Jakarta, Indonesia 10 College of Health Sciences, Addis Ababa University, Addis Ababa, Ethiopia 11 Mahidol-Oxford Tropical Medicine Research Unit, Faculty of Tropical Medicine, Mahidol University, Bangkok Thailand
**Name and contact information for the trial sponsor {5b}**	Menzies School of Health Research, Darwin, Australia
**Role of sponsor {5c}**	The study sponsor has full authority on study design, data collection, management, analysis, and interpretation of data, writing of the report, and the decision to submit the report for publication.

## Introduction

### Background and rationale {6a}

*Plasmodium vivax* forms dormant liver stages that can reactivate weeks or months following an acute infection. Recurrent infections are associated with a febrile illness, a cumulative risk of severe anaemia, direct and indirect mortality, and are an important source of onward transmission of the parasite. In co-endemic areas, up to 50% of patients re-present with *P. vivax* parasitaemia following treatment of acute *P. falciparum* malaria [1–3]. This is far greater than would be expected from reinfection alone. An association between the speed of initial parasite clearance and subsequent recurrent *P. vivax* suggests that relapses may be triggered by the febrile illness of acute malaria [2]. Hence, in co-endemic regions, there is a good rationale for opportunistically eradicating *P. vivax* hypnozoites from the liver in patients presenting with uncomplicated *P. falciparum* infections (Supplement 1).

The only hypnozoiticidal drug that is widely available is primaquine (PQ), an 8-aminoquinoline (8-AQ), which can cause severe drug-induced haemolysis in individuals with glucose-6-phosphate-dehydrogenase (G6PD) deficiency. The risk of haemolysis after PQ depends on the dose administered and the G6PD activity of the individual exposed. The risk of serious haemolysis associated with G6PD deficiency makes clinicians reluctant to prescribe PQ without prior testing, which is often unavailable [4]. When PQ is prescribed, patient adherence to the standard 14-day regimen is poor, resulting in a high risk of relapse. In some locations, the effectiveness of 14 days of unsupervised PQ is extremely poor [5, 6].

The recently completed multicentre IMPROV study compared the efficacy of a 7-day PQ regimen (1.0 mg/kg/day for 7 days) with a 14-day regimen (0.5 mg/kg/day for 14 days). PQ was given with either chloroquine (CQ) (in Ethiopia, Vietnam and Afghanistan) or Dihydroartemisinin-piperaquine (DHA-PIP) (in Indonesia). In total, 2388 patients were enrolled and screened for G6PD deficiency using the fluorescent spot test (FST). The 7-day PQ regimen (PQ7) was non-inferior to the 14-day regimen and 5-fold more efficacious at reducing *P. vivax* recurrence than the control (CQ or DHA-PIP alone) [7]. Whilst there were slightly more minor adverse events in the PQ7 arm, the regimen was generally well tolerated with no patients requiring blood transfusion. Adherence to and thus effectiveness of the shorter course of PQ is likely to be significantly better than that of the 14-day regimen.

There is a need for safe and effective radical cure of malaria in areas co-endemic for both *P. falciparum* and *P. vivax*, and the benefits of this treatment are likely to extend to patients presenting with uncomplicated malaria due to either species. The potential to provide a radical cure safely with a 7-day regimen of PQ provides major practical advantages. Our multicentre randomized, open-label trial compares the safety and efficacy of a high dose PQ treatment in G6PD normal patients with *P. falciparum* to reduce the risk of subsequent *P. vivax* episodes to the current standard practice of providing only schizonticidal treatment.

### Objectives {7}

#### Primary objective

To assess the safety and efficacy of a 7-day course of high dose PQ treatment regimen in preventing recurrent *P. vivax* parasitaemia by day 63 in G6PD normal patients following uncomplicated *P. falciparum* malaria.

#### Secondary objectives

- To assess the efficacy of a 7-day course of high dose PQ treatment regimen in preventing *P*. *falciparum* parasitaemia.
- To assess the efficacy of 7-day high dose PQ treatment in reducing gametocyte carriage of *P*. *falciparum*.
- To assess the safety and tolerability of high dose PQ in patients with *P. falciparum malaria*.

### Trial design {8}

This study is a multicentre, health care facility-based, randomized, controlled, open-label superiority trial comparing high dose PQ treatment to prevent *P. vivax* parasitaemia in patients presenting with uncomplicated *P. falciparum* mono-infection.

## Methods: participants, interventions and outcomes

### Study setting {9}

The study is a multicentre study carried out in study sites in Ethiopia, Bangladesh and Indonesia.

### Eligibility criteria {10}

#### Inclusion criteria for the pre-study

Participants must meet the following criteria to be eligible for the pre-study:

- Healthy male adult
- No blood transfusion or major surgery in the last 3 months
- Written informed consent

#### Inclusion criteria for the trial

Patients must meet the following criteria to be eligible for the trial:

- *P. falciparum* mono-infection
- Fever (axillary temperature ≥37.5 °C) or history of fever in the preceding 48 h
- Age >1 year (≥ 18 years at the Ethiopia site)
- G6PD normal as defined by the Biosensor (SD Bioline, ROK) at ≥70% of the adjusted male median (AMM) for each site
- Written informed consent
- Able to comply with all study procedures and timelines

#### Exclusion criteria for the trial

If the patients meet any of the following criteria at the screening, they will not be eligible for the trial:

- General danger signs or symptoms of severe malaria
- Anaemia, defined as Hb <8g/dl
- Pregnant women as determined by urine β-HCG pregnancy test
- Breastfeeding women
- Known hypersensitivity to any of the drugs given
- Regular use of drugs with haemolytic potential
- Blood transfusion within the last 4 months

### Who will take informed consent? {26a}

Patients attending one of the study health centres will be screened for enrolment into the trial. If patients are eligible and willing to participate in the study, they will be asked to sign a written informed consent form for enrolment. Written informed consent will be obtained by trained and authorized staff. In the case of minors, the legal guardian will be asked to sign on behalf of the minor. In addition, minors above the age of 11 years will be asked to provide written assent.

### Additional consent provisions for collection and use of participant data and biological specimens {26b}

In Ethiopia, patients will be asked for additional consent to provide up to 3 blood samples on day 5 within 1 to 6 h after treatment to determine drug levels. At the study site in Bangladesh, participants will be asked to provide additional consent for the collections of costs incurred by the patient during their illness to inform cost analysis. All patients will be asked to provide additional consent for the long-term storage of blood samples to conduct tests not included in the protocol. Those additional procedures will require additional ethical approval.

## Interventions

### Explanation for the choice of comparators {6b}

All participants in the control arm will be treated for acute uncomplicated falciparum malaria according to local guidelines. In Ethiopia and Bangladesh, this will be with artemether-lumefantrine (AL) administered twice daily over three days according to the manufacturer’s recommendations. In Indonesia, patients will be treated with Dihydroartemisinin-piperaquine (DHA-PIP) administered once daily over three days according to the manufacturer’s recommendations. If the participant vomits the treatment within 60 min, a repeat dose will be administered. If the patient vomits the second dose, the patient will receive second-line treatment. In the control arm, patients will be prescribed a single dose of PQ if this is included in the national guidelines for the eradication of *P. falciparum* gametocytes. All treatment will be directly supervised.

### Intervention description {11a}

All participants in the intervention arm will be treated for acute uncomplicated falciparum malaria according to local guidelines and as outlined for the control group. In addition, patients in the intervention arm will be administered a high-dose PQ regimen over 7 days (1.0 mg/kg/day for 7 days), which will be commenced on day 0 and administered along with a small snack. All treatment will be directly supervised.

### Criteria for discontinuing or modifying allocated interventions {11b}

Each participant has the right to withdraw from the study at any time. If the participant withdraws consent or is lost to follow-up for 4 or more weekly visits, the patient will be excluded/withdrawn. It will be left to the Investigator’s clinical judgement whether or not an adverse event (AE) is of sufficient severity to require stopping the participant’s treatment. If the participant is withdrawn due to an AE, the investigator will arrange for follow-up visits or telephone calls until the AE has resolved and conditions stabilized.

#### Rescue treatment

Patients who fail to respond adequately to the study treatment will be administered rescue treatment according to national guidelines. Patients who develop severe malaria will be admitted to hospital and treated with the recommended parenteral drug (intravenous or intramuscular artesunate or intramuscular artemether) according to national guidelines.

### Strategies to improve adherence to interventions {11c}

Patients will be asked to return to the health centre for directly observed treatment for all drug doses. If patients do not return all efforts will be made to restart treatment.

### Relevant concomitant care permitted or prohibited during the trial {11d}

At enrolment, all anti-malarial medication received in the preceding 4 weeks will be documented on the clinical record form (CRF). Regular medication at trial entry for conditions other than malaria, e.g. asthma, hypertension, etc., will be documented. Patients will be asked to continue to take these regular medications in the normal way. Any additional drugs taken during the trial period for whatever reason will be documented (e.g. antibiotics for inter-current infection, anti-emetics, anti-pyretics). Patients who discontinue their trial medication prematurely, or who fail to respond to trial medication and receive other anti-malarial therapy, will be recorded with a start and end date. Drugs with antimalarial activity should be avoided, unless prescribed by the attending clinician.

### Provisions for post-trial care {30}

The sponsor has insurance, which is in accordance with the legal requirements. This insurance provides coverage for damage to research subjects through injury or death caused by any activities of the study.

### Outcomes {12}

#### Efficacy outcomes

Primary: The incidence risk of any *P. vivax* parasitaemia on day 63

Secondary:

- The incidence risk of symptomatic *P. vivax* parasitaemia on day 63
- The incidence risk of any *P. vivax* parasitaemia on days 28 and 42
- The incidence risk of any *P. falciparum* malaria on days 28, 42 and 63
- The incidence risk of *P. falciparum* gametocytaemia between days 7 and 63
- Parasite clearance on days 1, 2 and 3
- Fever clearance on days 1, 2 and 3

#### Safety outcomes

- The proportion of patients vomiting their medication on the day of enrolment within 1 hour of administration;
- The proportion of patients vomiting any of their PQ doses within 1 hour of administration;
- The proportion of adverse events and serious adverse events;
- The incidence risk of severe anaemia (Hb<5g/dl) and moderately severe anaemia (<7g/dl) and/or the risk for blood transfusion between days 3 and 7;
- The incidence risk of ≥25% fall in haemoglobin since baseline with and without haemoglobinuria on days 3 and 7;
- The incidence risk of ≥25% fall in haemoglobin to under 7g/dl with and without haemoglobinuria on days 3 and 7.

### Participant timeline {13}

The participant timeline can be found in Supplement 2.

### Sample size {14}

This study was powered assuming that the risk of *P. vivax* after *P. falciparum* is highest after treatment with AL, which is the schizonticidal treatment used in two of the three study sites (Bangladesh and Ethiopia). Assuming a risk of *P. vivax* after *P. falciparum* infection on day 63 after AL treatment of 41% and a reduction of this risk to 20% in the intervention arm [1, 2, 8], a total sample size of 322 patients will have 98% power at the two-sided 5% significance level. Sample size calculations were performed using the power function in Stata, version 16.

Assuming a loss to follow up rate of 20%, the sample size will be increased to 403 across the sites in Ethiopia and Bangladesh where AL is used. We aim to recruit between 300 and 350 patients in Ethiopia, up to 50 in Bangladesh depending on the speed of recruitment.

We further aim to recruit 100 patients in Indonesia, which uses the schizonticidal treatment DHA-PIP. If we do not observe between site differences then estimates from the three sites will be combined giving >99% power for the primary outcome and additional power for the secondary outcomes.

### Recruitment {15}

Patients will be recruited at established clinical trial sites in malaria-endemic areas. Recruitment was initially planned over a period of 1 year but was extended due to COVID-19-related interruptions and halting of clinical trial recruitment.

## Assignment of interventions: allocation

### Sequence generation {16a}

Patients with malaria who are G6PD normal will be allocated randomly to one of two arms. The ratio of enrolment is 1:1 and randomization is done in blocks of 8.

### Concealment mechanism {16b}

Sealed envelopes are prepared by an independent statistician before the study starts. Individual envelopes will only be opened after the screening is completed for the patients qualified to be enrolled into the study.

### Implementation {16c}

Allocation sequence is generated by an independent statistician. Enrollment and assignment of participants is done by qualified study staff who have received delegation by the study PI to conduct these tasks.

## Assignment of interventions: blinding

### Who will be blinded {17a}

Neither participants nor study staff will be blinded to the allocation.

### Procedure for unblinding if needed {17b}

Since the study is not blinded no procedures for unblinding are required.

## Data collection and management

### Plans for assessment and collection of outcomes {18a}

#### Pre-study

Only patients with a G6PD activity ≥ 70% of the adjusted male median (AMM) as determined by the Biosensor (SD Bioline, ROK) will be eligible for enrolment into the main trial. In order to determine 100% G6PD activity at each site, a total of 30 adult males attending the health facility will be sampled. Only patients negative for malaria as confirmed by rapid diagnostic test (RDT) and without fever will be asked to participate. Separate written informed consent will be requested. A fingerpick sample (total volume 10 μl) will be collected and G6PD activity measured using the Biosensor. No follow-up of those participants will be required.

#### Study procedures for the main study

##### Day 0

For screening purposes, a finger-prick blood sample will be collected and used for malaria slide, Hb measurement and G6PD testing using the Biosensor. Women between the ages of 13 to 49 years willing to participate will be asked to take a urinary pregnancy test. Participants who have a G6PD deficient result will be informed of their status and will be provided with information on G6PD deficiency and its consequences in everyday life and consecutive malaria infections.

Eligible patients who provide written informed consent will be enrolled into the trial. Following enrolment, a brief questionnaire will be completed including demographic information, medical history, treatment history, history of recent blood transfusions, and a brief physical examination will be performed. 7.5 ml of venous blood will be collected in all consenting participants, and 450 μl of capillary blood will be collected in those who decline venous blood collection. Blood will be used for subsequent host and parasite analyses. All participants will receive the first dose of schizonticidal treatment under supervision. Patients in Bangladesh and Ethiopia who receive AL will be instructed to take the second dose at home. Patients in the intervention arm will receive their first dose of PQ.

Baseline (pre-dose) urinary sample (not more than 50 ml) will be collected from the participants (except for female participants age between 13 and 49 years old, whose urinary samples have been collected for pregnancy test purpose) before the administration of high-dose PQ. Post-dose urinary sample (not more than 50 ml) will also be collected from the participants in the intervention arm within 3–6 h following PQ administration.

Baseline (pre-dose) metHb level will be measured using a non-invasive Masimo MetHb device before the administration of high-dose PQ. The metHb level will also be measured following the dosing of PQ with 30-min interval up to 4 h (Indonesia site only).

##### Day 1

All participants will be reviewed and the next dose of schizonticidal treatment will be provided. Patients in Bangladesh and Ethiopia who receive AL will be instructed to take the second daily dose at home. Patients in the intervention arm will receive their second dose of PQ. Capillary blood will be collected, a malaria smear prepared and Hb measured.

##### Day 2

All participants will be reviewed and treated with schizonticidal treatment under supervision. Patients in Bangladesh and Ethiopia who receive AL will be instructed to take the second daily dose at home. Patients randomized to the intervention arm will receive their third dose of PQ treatment under observation. Capillary blood will be collected, a malaria smear prepared and Hb measured. The remaining sample will be used to assess G6PD activity using the Biosensor.

##### Days 3–6

Participants in the intervention arm will be reviewed daily and treated with supervised PQ. Participants will be screened for any evidence of haemoglobinuria (dark urine) or adverse symptoms. If there are clinical concerns of haemolysis, a capillary blood sample will be taken for Hb measurement. An aliquot of the sample on day 6 will also be used to confirm drug levels. Additional more intense sampling for drug levels will be done on day 5 in Ethiopia only. For those patients, a quantitative G6DP test using the Biosensor will also be done. Patients in the control arm will not have scheduled visits before day 7.

##### Days 7, 14, 21, 28, 35, 42, 49, 56 and 63

All participants will be reviewed again on day 7 and weekly thereafter, or on any day when they have further symptoms, until day 63. A symptom questionnaire and a brief physical exam will be performed. A capillary blood sample will be collected for immediate Hb measurement and blood film examination. The remaining capillary blood will be stored in a microtainer. On days 7, 28 and 63, the same sample will be used for repeat testing of G6PD activity using the Biosensor. No more than 450 μl of capillary blood will be collected at each visit. In Indonesia, not more than 3 ml of venous blood will be collected on day 63.

##### Day of recurrence

All participants with fever or symptoms indicative of malaria will be asked to return to the same health centre as on enrolment. A symptom questionnaire will be completed, a brief physical examination undertaken, and a blood film will be examined for malaria diagnosis (which will be read immediately). In those patients diagnosed with recurrent parasitaemia, a 7.5-mL (1 tube) venous blood sample will be collected, Hb concentration will be measured, and the remaining blood will be stored in an EDTA tube for further analysis.

### Plans to promote participant retention and complete follow-up {18b}

All patients will be counselled before enrolment about the requirements during the trial, in particular about the frequency of follow up visits. The importance of completing the follow up will be highlighted, but patients will also be reassured that they can drop out of the study at any time without providing a reason.

### Data management {19}

Menzies School of Health Research, as a legal trial sponsor, will be responsible for data collection, storage, protection, retention and destruction. Source data will be collected on paper case record forms (CRF) which will be stored securely on site. The data will only be accessible to the investigators, research sponsors, study monitor, the auditor(s), regulatory authorities, ethics committee and delegated members of the study team, who will enter all clinical data. Patient data will be recorded in pseudonymized form (i.e. without reference to the patient’s name) using exclusively the patient’s identification code.

Data will be entered into the online database REDCap by users assigned individual usernames and passwords. The database includes range checks for data values and internal validation checks. In addition, logic checks will be performed as well as 10% random checks at each monitoring visit to ensure data correctness and integrity. The database will be closed, data extracted and stored on WorldWide Antimalarial Resistance Network (WWARN.org) servers. The WWARN is registered with the Registry of Research Data Repositories (re3data.org).

### Confidentiality {27}

All study participant information will be stored using unique identifiers. The key to the identification code list will only be available to the immediate study site team and only during the study. No identifying patient details will be reported to anyone outside the study team or at the publication stage.

### Plans for collection, laboratory evaluation and storage of biological specimens for genetic or molecular analysis in this trial/future use {33}

#### Sample collection

Microtainer™ samples will be collected by finger prick on scheduled and unscheduled visits. At each visit no more than 450 μl will be collected, to a total volume of 5 ml. Red blood cell (RBC) pellets and plasma will be separated by centrifugation and stored at − 80°C and − 20°C, respectively. 7.5 ml of EDTA-anticoagulated venous blood samples will be collected on D0 and day of recurrence. In the case of infants and children, a total of 0.5 ml/kg up to a total of 7.5 ml will be collected. The sample will be white blood cell (WBC)-depleted and both RBC pellets and plasma stored at − 80°C and − 20°C, respectively, for further laboratory analyses. A total volume of 12.5–20 ml blood will be collected within 63 days; this volume is well within the WHO guidelines for clinical trials.

#### Malaria microscopy

Slides for microscopy will be collected on all scheduled and unscheduled visits. Slides will be read immediately. Quality control and assurance will be performed according to standard SOPs.

#### Haemoglobin

Hb will be measured in all patients at enrolment, days 1 and 2. In the intervention group, Hb will also be measured on any day between days 3 and 6 when clinically warranted. Subsequently, Hb will be measured weekly from day 7 onwards. Hb will be measured using a Hemocue™ Hb (Angelholm, Sweden) machine or another rapid diagnostic test such as the Carestart Hb machine (Carestart, USA).

#### G6PD deficiency testing

One hundred percent G6PD activity at each site will be determined before the start of the study using Biosensor (SD-Bioline, ROK) readings from 30 male adults.

In the trial, G6PD activity will be measured quantitatively using the Biosensor on day 0 for screening. Further Biosensor readings will also be done on days 2, 7, 28 and 63 and opportunistically if blood is collected in the intervention arm between days 3 and 6.

#### Urine β-HCG pregnancy test

A urine β-HCG pregnancy test will be conducted on all women aged 13–49 years who are screened for the study. Anyone testing positive will be excluded from the study.

#### Cell-free Hb

Cell-free Hb (CFHb) will be analysed on samples collected on day 0. For this purpose, whole blood samples will be double spun to remove platelets. Samples will then be stored frozen and further processing will be done at the Menzies School of Health Research, Darwin, Australia.

#### Parasite biomass

LDH/HRP2 ELISA assays for the assessment of the total biomass will be done from samples collected on day 0 and the day of recurrence. Plasma samples will be stored frozen and further processing will be done at the Menzies School of Health Research, Darwin, Australia.

#### Parasite molecular analysis

Sub-patent infections (PCR positive, but blood film negative) are common and can result in anaemia, increased risk of recurrent symptomatic parasitaemia, and ongoing transmission. Parasite DNA will be extracted from RBC pellets for PCR analysis. PCR will be undertaken for speciation and quantitation of sub-patent infections and genotyping. All *Plasmodium* spp. detected will be typed for genetic fingerprinting (to determine new or different parasites from previous or later infections), and known and putative molecular markers of drug resistance*.* Whole-genome sequencing and molecular typing analyses of parasites will be done by using new-generation high throughput platforms (i.e., Illumina, Sequenom) at the Sanger Institute, UK, or similar techniques at other facilities.

#### Host genotyping and RBC polymorphisms

Human DNA will be extracted from collected samples. All samples will be assessed using molecular techniques for known and unknown variants of the G6PD gene (Xq28) and other candidate markers related to malaria susceptibility and treatment outcomes such as alpha and beta thalassaemia, Gerbich and Ovalocytosis.

Red blood cell polymorphisms will be assessed by molecular analysis. CYP2D6 polymorphisms which are known to affect PQ metabolism will also be determined by host genotyping. Where possible, all tests will be undertaken in-country. However, where the laboratory does not have the capacity, samples will be transferred to a reference laboratory.

#### Drug concentrations

Blood collected on day 6 and day of recurrence will be used to measure the plasma concentrations of PQ and its metabolites (such as carboxyprimaquine), piperaquine, lumefantrine and desethyl-lumefantrine. Day 6 concentrations will be correlated with the risk of *P. vivax* recurrence. In addition, in Ethiopia (with additional consent) up to 3 random samples 1 to 6 h post-treatment will be collected on day 5 to collect detailed pharmacokinetic (PK) data. Samples will be capillary samples collected into microtainers (max 100 μl per sample). Drug concentrations will be measured at the Mahidol Oxford Research Unit, Bangkok, Thailand, or an institution with a similar capacity.

#### Serology

Blood collected from capillary or venous sampling on baseline, days 7, 28 and 63 will be used for serological analysis to quantify antibody levels and complement fixation and to assess how these are correlated with treatment efficacy. Antibody levels against other infectious diseases (except HIV) that may be associated with malaria immune responses will be measured. Samples will be processed on site and stored at − 80°C and shipped on dry ice to the Menzies School of Health Research, Darwin, Australia.

#### Meth-haemoglobin

Methylated Hb levels will be measured in patients at the Indonesian study site only. A non-invasive monitoring platform (Masimo) will be used. Measurements will be taken by placing a sensor on the fingertip of the patient. The measurements will be taken just before the daily PQ dose and continued up to approximately 4 h in 30-min intervals making a total of 9 measurements in total for 3 days.

## Statistical methods

### Statistical methods for primary and secondary outcomes {20a}

To provide a pragmatic comparison of the different drug treatments, the principle of intention-to-treat (ITT) will be the main strategy of analysis adopted for the primary and secondary endpoints. These analyses will be conducted on all patients assigned to the treatment groups as randomized, regardless of the study treatment received. An analysis based on a per-protocol (PP) approach will be conducted following the causal framework presented in Hernan & Robins [9]. The following participants will be excluded from the PP analysis:

- Ineligibility (either arising during the study or retrospectively having been overlooked at screening)
- Significant protocol deviation (e.g., given a wrong total dose of PQ)
- Incomplete treatment course

A causal diagram will be drawn to identify any pre- or post-randomization confounders to guide the analysis approach.

The safety data will be analysed using a safety data population comprising all patients who received at least one dose of treatment.

The incidence risk (95% CI) of *P. vivax* parasite recurrence within 63 days of follow-up (primary outcome) will be calculated using the Kaplan-Meier (KM) method for each trial arm as well as a comparison of the relative hazards between trial arms (hazard ratio (95% CI)) estimated from a Cox regression model for the time to the first recurrent episode with stratification for study site*.* The first episode of *P. vivax* (including mixed infections with *P. vivax*) will be treated as a failure endpoint. Patients presenting with parasitaemia other than *P. vivax* will be censored at the day of occurrence.

Comparisons will be made between the intervention and control arm. The analyses will be performed in the ITT and the PP population with stratification for the site. The same analysis approach will be used for the secondary outcome measures: symptomatic *P. vivax* parasitaemia on day 63; any *P. vivax* parasitaemia on days 28 and 42; any *P. falciparum* parasitaemia on days 28, 42 and 63; and any *P. falciparum* gametocytaemia on days 7 and 63.

For safety data, the proportion and number of adverse and serious adverse events will be presented by intervention and control arms. Values and changes from baseline especially for Hb will be calculated for each visit by the treatment group.

More details on the analysis of primary and secondary outcomes and safety analyses are provided in Supplement 3.

### Interim analyses {21b}

No formal interim analysis is planned, unless otherwise advised by the Data Safety Monitoring Board (DSMB).

### Methods for additional analyses (e.g. subgroup analyses) {20b}

No a priori subgroup analyses are planned.

### Methods in analysis to handle protocol non-adherence and any statistical methods to handle missing data {20c}

Patients can have an incomplete course of treatment or data on drug administration may be missing. No imputation of treatment courses will be made for patients with missing data. For patients with missing data on AEs, the most conservative approach will be used. Rules for the adjudication of microscopy related endpoints as well as safety endpoints can be found in the Statistical Analysis Plan (Supplement 3).

### Plans to give access to the full protocol, participant-level data and statistical code {31c}

The dataset will be uploaded in a repository such as the WWARN data repository or other suitable databases.

## Oversight and monitoring

### Composition of the coordinating Centre and trial steering committee {5d}

This is a multicenter study coordinated by the Menzies School of Health Research, Darwin, Australia, with study sites in Bangladesh managed by the icddr,b, in Ethiopia managed by Arba Minch University and in Indonesia managed by EOCRU. Day-to-day support for all trial sites is provided by the Menzies based study team including (i) the principal investigator, responsible for the overall trial conduct, (ii) the data manager, who is responsible for database development and maintenance, (iii) the study coordinator, responsible for trial registration, training of study staff on site, monitoring and logistical issues as well as annual reporting and (iv) the statistician, responsible for data analysis (note, the randomization was performed by an independent statistician).

No trial steering committee has been appointed.

### Composition of the data monitoring committee, its role and reporting structure {21a}

A DSMB has been appointed for this study. The DSMB is responsible for safeguarding the interests of study participants, assessing predominantly the safety of study procedures, and for monitoring the overall conduct of the study. The DSMB members are independent and it is their responsibility to prevent patients being exposed to any excess risks by recommending trial suspension or termination early if the safety or efficacy results are sufficiently convincing. The responsibilities of the DSMB are (i) to determine how frequently interim reviews of trial data should be undertaken, (ii) to conduct interim safety data reviews, and (iii) to report (following each DSMB meeting) to the sponsor and to recommend whether the trial should continue, the protocol be modified or the trial be stopped.

Reports of specific serious adverse events (SAEs) are sent for review to the DSMB as they occur. This includes SAE reports of patients with acute haemolysis defined as Hb<7g/dl, anyone requiring blood transfusion or macroscopic haemoglobinuria (Hillmen ≥5) plus a fractional fall in Hb ≥25% from baseline, independent of the study arm. This will also include any SAEs which the investigator categorized as possibly or definitely related to the study drug.

Quarterly reports are sent to the DSMB for review including (i) all SAE reports; (ii) line listings of all adverse events stratified by severity and study arm; (iii) a flow diagram with the number of screened and enrolled patients, patients who have been withdrawn or lost to follow-up; (iv) line listing of all protocol violations, v) individual patient Hb data between day 0 and day 14; (vi) the number of patients with fall in Hb ≥ 25% or reporting microscopic haemoglobinuria.

Further details about the DSMB charter may be obtained upon reasonable request.

### Adverse event reporting and harms {22}

#### Procedures for serious adverse events (SAEs)

All participants with SAEs will be reviewed with a standard questionnaire. Patients suspected of severe malaria or sepsis will be treated according to local hospital guidelines.

All patients with an SAE will be assessed to ascertain malaria and haematological status. Relevant investigations will be ordered according to the attending physician. All results of clinical investigations will be collated on an SAE report form.

All SAEs will be reported by the site investigator to the study coordinator within one working day of awareness, who will, in turn, notify the DSMB and PI within 48 h of their notification of the event. The site PI will be responsible for notifying the local Ethical Committees as appropriate. All information received for a case will be detailed on a full SAE report form.

Adverse events will be recorded for up to 42 days after the last day of administration of PQ. All related AEs that result in a participant’s withdrawal from the study, or are present at the end of the study, will be followed up until a satisfactory resolution occurs. It will be left to the Investigator’s clinical judgement whether or not an AE is of sufficient severity to require stopping the participant’s treatment. A participant may also voluntarily withdraw from treatment due to what he or she perceives as an intolerable AE. If either of these occurs, the participant must undergo an end of study assessment and be given appropriate care under medical supervision until symptoms cease or the condition becomes stable.

An increase of >20% in metHb level of a subject will be recorded as an AE and the subject will be closely monitored. The subject will receive the treatment of acute methemoglobinemia according to the increase and their clinical features (Indonesia only).

#### Management of patients with anaemia related adverse events

Malaria can cause a temporary fall in Hb that can be exacerbated by PQ. All patients will be monitored closely for acute recovery from their initial episode of malaria, any adverse drug reactions, and subsequent recurrence of malaria. In patients suspected of acute haemolysis, the clinical judgement of the attending clinician and patient safety should always come first, regardless of the trial protocol.

### Frequency and plans for auditing trial conduct {23}

A detailed monitoring plan has been prepared. In brief, it states that a minimum of 2 monitoring visits (virtual or in-person) will be conducted during the course of the study. If virtual monitoring, each monitoring visit will take place using online video conferencing and secure, encrypted file sharing. No identifying information will be stored on the server. Monitoring frequency may be increased based on the following criteria: (i) high subject enrolment at a particular site, (ii) compliance issues (e.g. significant protocol violations), (iii) site staff turnover, requiring additional training, and (iv) results and findings from previous monitoring visit. Detailed procedures are captured in the monitoring plan, which can be requested. Auditing can also take place by national health authorities at the participating study sites.

### Plans for communicating important protocol amendments to relevant parties (e.g. trial participants, ethical committees) {25}

Changes to protocol modification will be communicated promptly to all involved parties and updated in the clinical trials registry. Any protocol deviations will be fully documented.

### Dissemination plans {31a}

Results will be published in peer-reviewed journals. All Investigators will be involved in reviewing drafts of the manuscripts, abstracts, press releases, and any other publications arising from the study. Authorship will be determined in accordance with the ICMJE guidelines and other contributors will be acknowledged. Results will further be disseminated at international conferences and other stakeholder meetings including National Malaria Control programs in the region. All efforts will be made to disseminate results to study participants in line with local requirements.

## Discussion

Recurrent episodes of *P. vivax* put a strain on healthcare systems. A unified treatment policy for malaria has potential to confer significant individual, public health and operational benefits in regions co-endemic for *P. falciparum* and *P. vivax*. The optimal use of limited financial resources could necessitate strategies for treating *P. vivax* through universal radical cure.

## Trial status

Recruitment started on 18 August 2019 at the study site in Bangladesh and has been completed. The trial was paused in 2020 due to the COVID19 pandemic. Recruitment at the study in Ethiopia started on 27 January 2021 and on 1 June 2021 at the Indonesian site. The study is anticipated to be completed in 2022. The current protocol version number is V6.0.

## Supplementary Information

**Additional file 1.** Rationale for study.

**Additional file 2.** Participant timeline.

**Additional file 3.** Statistical Analysis Plan.

## Abbreviations

PQ: Primaquine
8-AQ: 8-aminoquinoline
G6PD: Glucose-6- phosphate-dehydrogenase
FST: Fluorescent spot test
CQ: Chloroquine
DHA-PIP: Dihydroartemisinin-piperaquine
Hb: Haemoglobin
AL: Artemether-lumefantrine
CRF: Case record form
WWARN: WorldWide Antimalarial Resistance Network
WBC: White blood cell
RBC: Red blood cell
CFHb: Cell-free Hb
