# Supplementary material for: Reducing the risk of Plasmodium vivax after falciparum infections in co-endemic areas—a randomized controlled trial (PRIMA)
Source: Trials. 2022 May 18;23:416. doi: 10.1186/s13063-022-06364-z (PMC9116071; doi:10.1186/s13063-022-06364-z)

Supplementary file 1: Rationale for study

**Evidence of the high risk of *P. vivax* following treatment of uncomplicated *P. falciparum***

There is a growing body of evidence highlighting a very high risk of *P. vivax* parasitaemia following treatment of *P. falciparum* infections; far higher than would be expected from the risk of reinfection alone. Even with highly effective ACTs, the greatest risk of recurrence after falciparum malaria is *P. vivax*, rather than *P. falciparum.* In co-endemic areas, patients presenting with *P. falciparum* have a high probability of having had prior infections with *P. vivax*, which may be present either at undetectable levels in the peripheral blood, or lying dormant in the liver. Both fever and/or haemolysis have been hypothesised to stimulate the reactivation of the dormant *P. vivax* liver stages resulting in relapsing infections. The risk is greatest with antimalarial agents with short elimination half-lives which offer little post-treatment prophylaxis to suppress the first relapse.

This provides a strong rationale for universal use of radically curative courses of PQ in patients with both *P. falciparum* and *P. vivax* malaria in areas that are co-endemic for these species. This proposition is supported by preliminary analysis demonstrating a reduced risk of *P. vivax* after *P. falciparum* in patients treated with supervised 14-day regimen of PQ (OR=0.48). It is important to note that the actual anti-relapse intervention is well proven and that the rationale is to extend this indication to other patient groups at high risk of recurrent *P. vivax*.

**Current evidence:**

1. Pooled analysis of risk of *P. vivax* parasitaemia in 10,549 patients presenting with *P. falciparum* malaria. *Douglas et al. Plasmodium vivax recurrence following falciparum and mixed species malaria: risk factors and effect of antimalarial kinetics. Clin Infect Dis 2011;* ***52****(5): 612-20.*
2. Systematic Literature review of 153 *P. falciparum* efficacy studies enrolling 31,262 patients from 323 site-specific treatment arms. *Commons et al. Risk of Plasmodium vivax parasitaemia after P. falciparum infection: a systematic review and meta-analysis. Lancet Infectious Diseases. Lancet Infect Dis. 2019 Jan;19(1):91-101.*
3. A WWARN individual patient data meta-analysis of 14,146 patients with *P. falciparum* from 10 countries treated with ACTs. *Hossein et al. The risk of Plasmodium vivax parasitaemia after P. falciparum malaria: An individual patient data meta-analysis from the WorldWide Antimalarial Resistance Network.* *PLoS Med. 2020 Nov 19;17(11):e1003393.*
4. A cluster randomised controlled trial in Papua Indonesia of DHA-piperaquine plus 14 days PQ for the treatment of both *P. vivax* and *P. falciparum* (TRIPI Study*). Poespoprodjo et al. Supervised versus unsupervised primaquine radical cure for the treatment of falciparum and vivax malaria in Papua, Indonesia: a cluster-randomised, controlled, open-label superiority trial.* *Lancet Infect Dis. 2022 Mar;22(3):367-376.*
5. ***Douglas et al.; Plasmodium vivax recurrence following falciparum and mixed species malaria: risk factors and effect of antimalarial kinetics. Clinical Infectious Diseases 2011; 52(5): 612-20.*** And accompanying editorial by Baird CID 2011.

A retrospective pooled analysis of 10,549 patients with uncomplicated malaria treated on the Thai-Myanmar border. The cumulative proportion of patients with *P. vivax* infection recurrence was 31.5% by day 63. Significant risk factors for *P. vivax* recurrence were mixed infection at enrolment, male sex, younger age, lower haematocrit, and higher asexual *P. falciparum* parasite density. By day 63, the cumulative risk of vivax malaria was 51.1% after treatment with rapidly eliminated drugs (artesunate), 35.3% after treatment with intermediate half-life drugs (AL or malarone), and 19.6% after treatment with slowly eliminated drugs (i.e., Mas3, DHA-Pip).


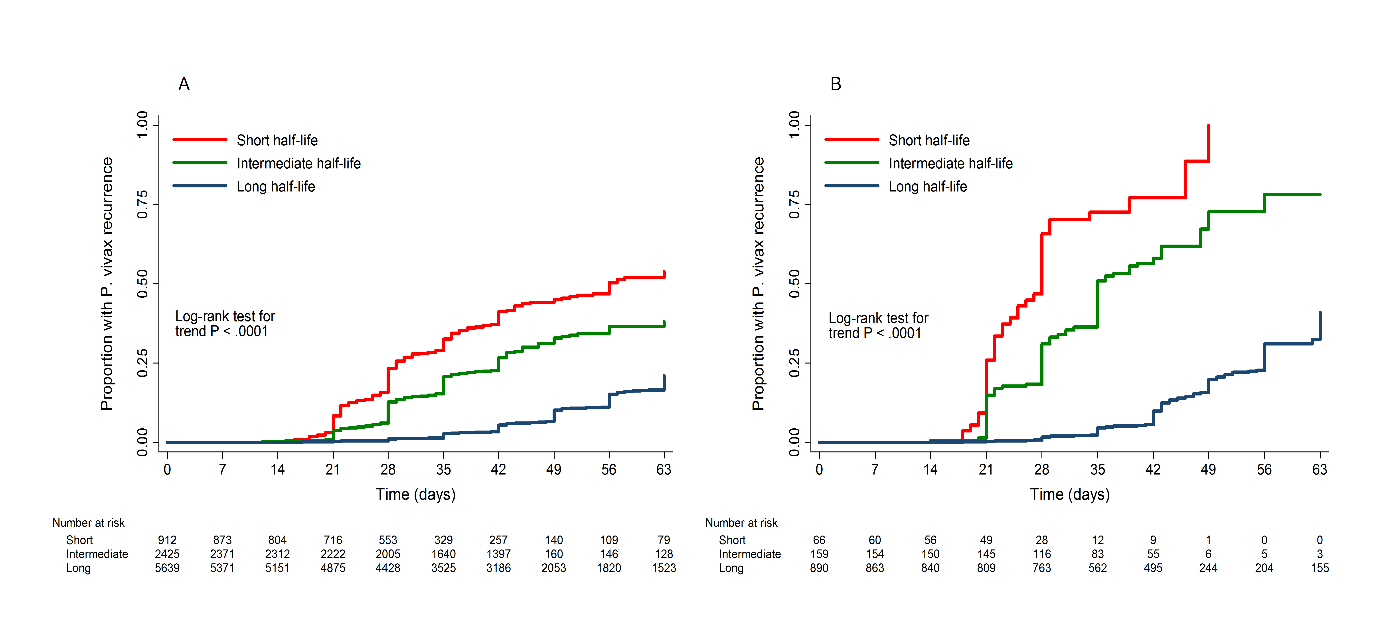


**Figure 1.** Risk of *P. vivax* recurrence after pure *P. falciparum* (A) and mixed species infection (B), following treatment with artemisinin combination therapy with fast and slowly elimination antimalarials.

1. ***Commons et al., Risk of Plasmodium vivax parasitaemia after P. falciparum infection: a systematic review and meta-analysis. Lancet Infect Dis. 2019 Jan;19(1):91-101.***

In total, 153 *P. falciparum* efficacy studies were included, enrolling 31,262 patients from 323 site-specific treatment arms: 130 (85%) studies were from the Asia-Pacific, 16 (10%) from The Americas, and 7 (5%) from Africa. The day 42 risk of *P. vivax* parasitaemia was 5·6% (95%CI 4·0-7·4; *I^2^*=92·0%; 117 estimates). The risk of *P. vivax* parasitaemia was greater in regions of short relapse periodicity and after more rapidly eliminated artemisinin-based combination therapy (ACT); reaching 15·3% (5·1-29·3; 97·2%; 10 estimates) after artemether-lumefantrine. Compared to artemether-lumefantrine, ACTs with mefloquine or piperaquine delayed recurrence, but by day 63, the risk of vivax parasitaemia was >15% for all ACTs assessed.


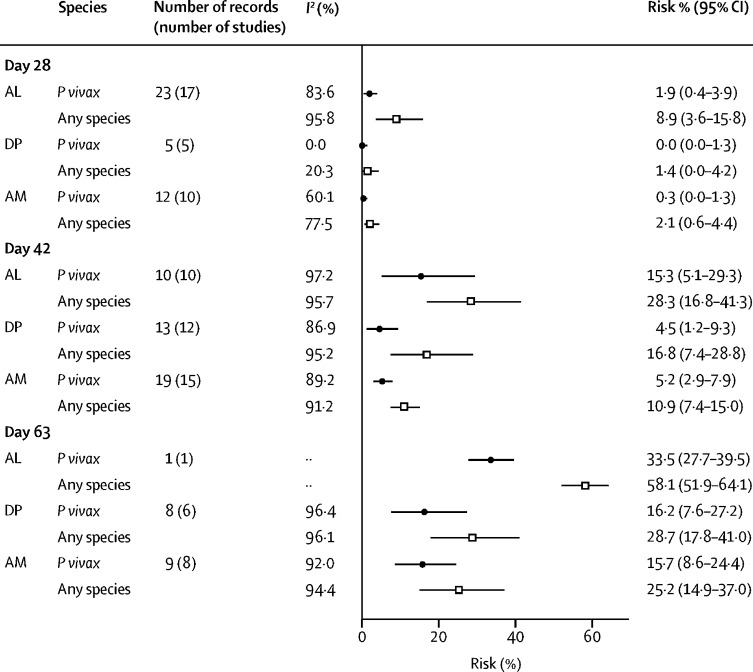


**Figure 2.** Risk of *Plasmodium vivax* parasitaemia or any parasitaemia *after Plasmodium falciparum* infection by artemisinin-based combination therapy and day of follow-up

Risk is the percentage of patients with *P. vivax* parasitaemia or any parasitaemia. AL=artemether-lumefantrine. DP=dihydroartemisinin-piperaquine. AM=artesunate-mefloquine.

1. ***Hossein et al. The risk of Plasmodium vivax parasitaemia after P. falciparum malaria: An individual patient data meta-analysis from the WorldWide Antimalarial Resistance Network.* *PLoS Med. 2020 Nov 19;17(11):e1003393.***

In total, 42 studies enrolling 15,341 patients were included in the analysis, including 30 randomised controlled trials and 12 cohort studies undertaken to determine efficacy of ACTs against *P. falciparum* in areas co-endemic for *P. falciparum* and *P. vivax*. Overall, 14,146 (92.2%) patients had *P. falciparum* monoinfection. In multivariable analyses, the highest rate of *P. vivax* parasitaemia over 42 days of follow-up was in patients residing in areas of short relapse periodicity (adjusted hazard ratio [AHR] = 6.2, 95% CI 2.0-19.5; p = 0.002); patients treated with AL (AHR = 6.2, 95% CI 4.6-8.5; p < 0.001), AA (AHR = 2.3, 95% CI 1.4-3.7; p = 0.001), or AM (AHR = 1.4, 95% CI 1.0-1.9; p = 0.028) compared with DP; and patients who did not clear their initial parasitaemia within 2 days (AHR = 1.8, 95% CI 1.4-2.3; p < 0.001).


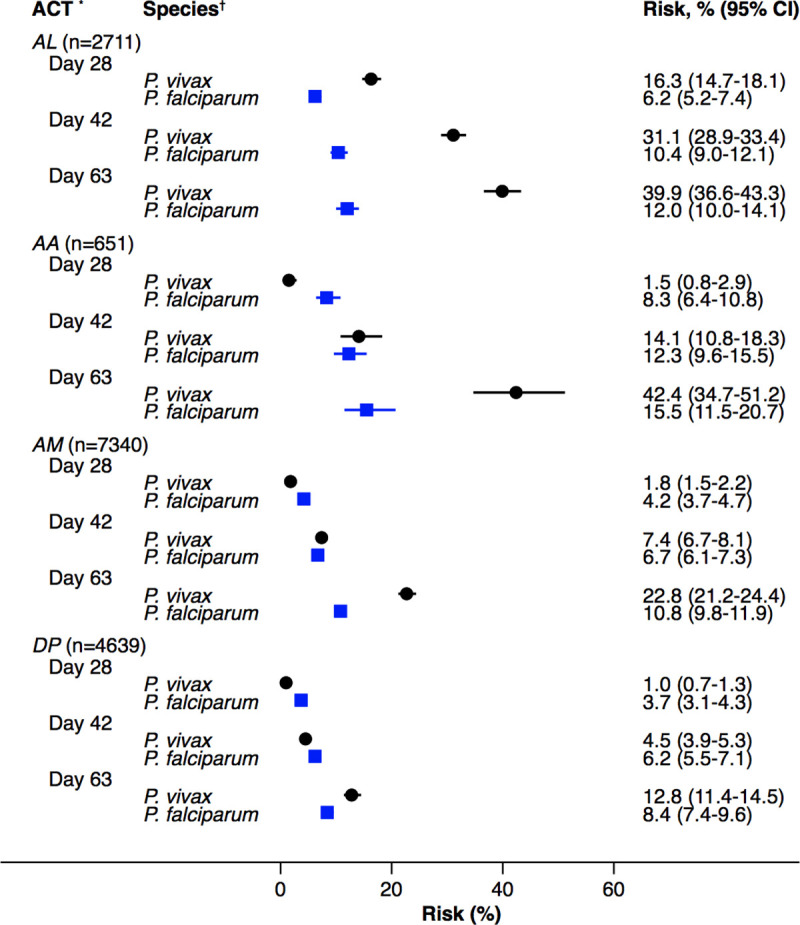


**Figure 2:** Cumulative risk (Kaplan–Meier analysis) of P. vivax parasitaemia following ACTs. *ACTs (AA, AL, AM, DP). †P. vivax recurrence includes recurrences with P. vivax monoinfection or mixed-species infection. AA, artesunate-amodiaquine; ACT, artemisinin-based combination therapy; AL, artemether-lumefantrine; AM, artesunate-mefloquine; DP, dihydroartemisinin-piperaquine.

1. ***Poespoprodjo et al. Supervised versus unsupervised primaquine radical cure for the treatment of falciparum and vivax malaria in Papua, Indonesia: a cluster-randomised, controlled, open-label superiority trial.* *Lancet Infect Dis. 2022 Mar;22(3):367-376.***

A cluster randomized, controlled, open label trial to assess the effectiveness of unsupervised versus supervised PQ treatment in patients with uncomplicated malaria in21 clusters each recruiting 20 patients with uncomplicated malaria (either P*. falciparum*, *P. vivax* or mixed infections). All patients were followed for 6 months. The study was conducted in Mimika, in the southern part of Papua Province, Indonesia, and completed in April 2018.

Two treatment arms:

- DHA-Piperaquine plus Primaquine which was supervised on alternate days
- DHA-Piperaquine plus Primaquine (0.5mg/kg/day) for 14 days started on day 2, unsupervised

Pill counts on day 16 in the unsupervised arm revealed a higher than expected adherence to treatment in the unsupervised treatment arm, which was hypothesised to be due to the enrolment process and intensive early follow up until day 3.

At the end of each cluster cohort, an additional 20 patients were enrolled and treated with standard policy with minimal patient interaction until day 14. Patients with *P. falciparum* were treated with DHA-Piperaquine plus a single dose Primaquine (PQ). Patients with *P. vivax* were treated with DHA-Piperaquine plus 14 days PQ.

The incidence rate for *P. vivax* recurrence was 539 (95% CI 390-747) infections per 1000 person-years in the supervised group versus 859 (673-1096) in the unsupervised group (incidence rate ratio 0·63 [95% CI 0·42-0·94]; p=0·025). The corresponding rates in the 224 patients who presented with *P. falciparum* malaria were 346 (95% CI 213-563) and 660 (446-977); incidence rate ratio 0·52 [95% CI 0·28-0·98]; p=0·043).


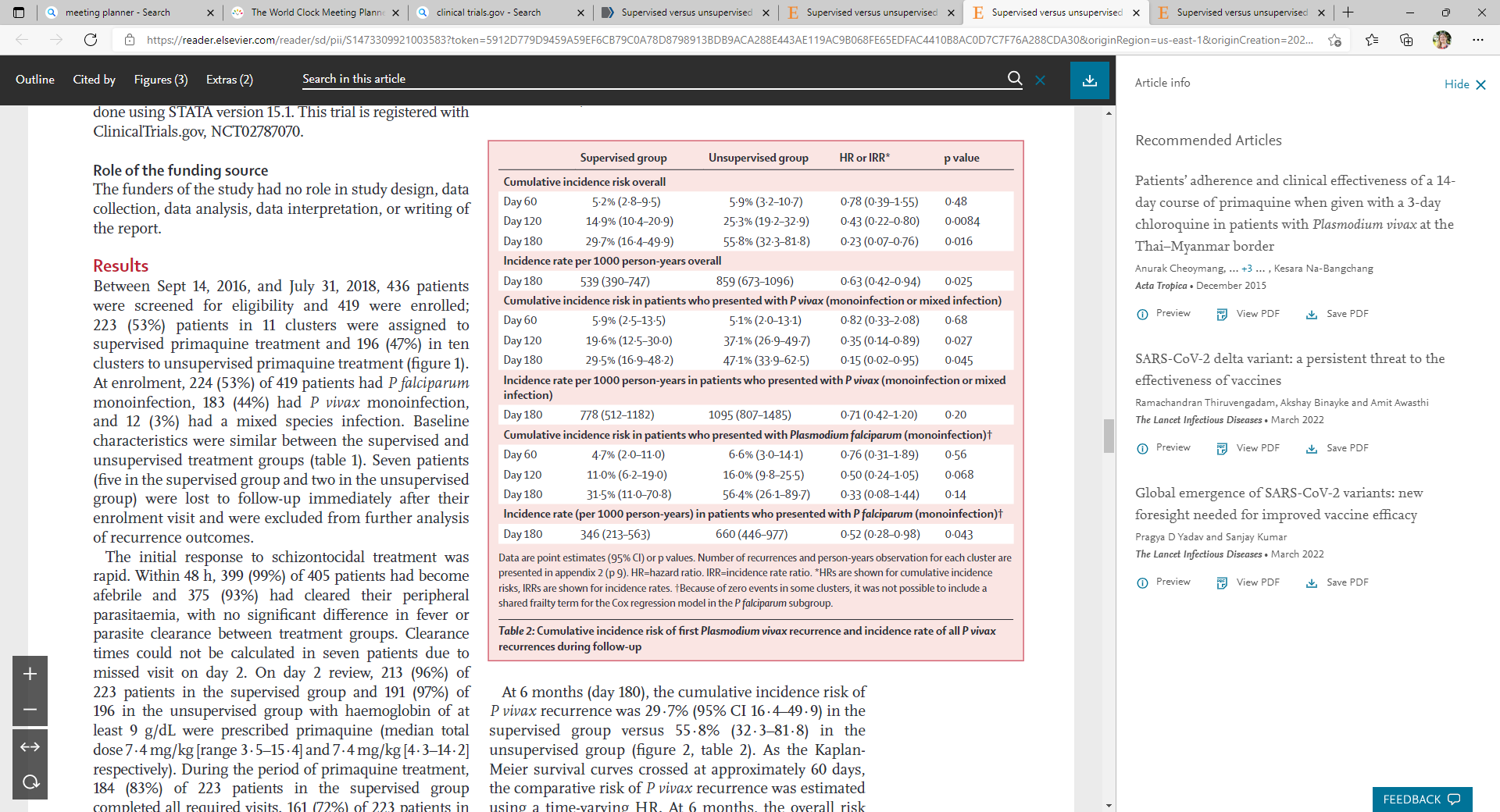

Supplement: Supplementary file 1 — Additional file 1. Rationale for study. [file 13063_2022_6364_MOESM1_ESM.docx]
