# Supplementary material for: Reducing the risk of Plasmodium vivax after falciparum infections in co-endemic areas—a randomized controlled trial (PRIMA)
Source: Trials. 2022 May 18;23:416. doi: 10.1186/s13063-022-06364-z (PMC9116071; doi:10.1186/s13063-022-06364-z)
Supplement: Supplementary file 2 — Additional file 2. Participant timeline. [file 13063_2022_6364_MOESM2_ESM.docx]

| **Time point after Enrolment** | **D0** | **D1** | **D2** | **D3-6^1^** | **7** | **14** | **21** | **28** | **35** | **42** | **49** | **56** | **63** | **Day of recurrence** |
| --- | --- | --- | --- | --- | --- | --- | --- | --- | --- | --- | --- | --- | --- | --- |
| **Treatment** |  |  |  |  |  |  |  |  |  |  |  |  |  |  |
| **Schizonticidal treatment** | X | X | X |  |  |  |  |  |  |  |  |  |  |  |
| **Primaquine treatment^1^** | X | X | X | X |  |  |  |  |  |  |  |  |  |  |
| **Procedures** |  |  |  |  |  |  |  |  |  |  |  |  |  |  |
| **Symptom Questionnaire** | X | X | X | X^2^ | X | X | X | X | X | X | X | X | X | X |
| **Medical Examination** | X | X | X | X | X | X | X | X | X | X | X | X | X | X |
| **Pregnancy test** | X |  |  |  |  |  |  |  |  |  |  |  |  |  |
| **Capillary blood collection (450 µl)** | X^5^ | X | X | X^3^ | X | X | X | X | X | X | X | X | X | X^5^ |
| **Venous blood collection (7.5 ml)^6^** | X |  |  |  |  |  |  |  |  |  |  |  |  | X |
| **G6PD biosensor** | X |  | X | X^4^ | X |  |  | X |  |  |  |  | X^9^ |  |
| **Malaria microscopy** | X | X | X |  | X | X | X | X | X | X | X | X | X | X |
| **Hb** | X | X | X | X^3^ | X | X | X | X | X | X | X | X | X | X |
| **Cell free Hb** | X |  |  |  |  |  |  |  |  |  |  |  |  |  |
| **Meth Hb^8^** | X |  |  |  |  |  |  |  |  |  |  |  |  |  |
| **Biomass** | X |  |  |  |  |  |  |  |  |  |  |  |  | X |
| **Drug levels** | X |  |  | X^7^ |  |  |  |  |  |  |  |  |  | X |
| **Parasite PCR/Genotyping** | X |  |  |  |  |  |  |  |  |  |  |  |  | X |
| **Host genotyping & RBC polymorphism** | X |  |  |  |  |  |  |  |  |  |  |  |  |  |
| **Serology** | X |  |  |  | X |  |  | X |  |  |  |  | X |  |
| 1 Intervention arm only  2 Day 3 only  3 Only if clinically warranted  4 Only if blood sample is collected for measurement of Hb  5 Only if no venous blood is collected  6 In case of infants and children a total of 0.5 ml/kg up to a total of 7.5 ml will be collected  7 3 random samples 1-6 hours post treatment on day 5 (Ethiopia only) and one pre-treatment samples on day 6 in the intervention group only  8 9 measurements over 4 hours every 30 min (Indonesia only)  9 3ml venous sample for G6PD(Indonesia only) | | | | | | | | | | | | | | |
