# Supplementary material for: Reducing the risk of Plasmodium vivax after falciparum infections in co-endemic areas—a randomized controlled trial (PRIMA)
Source: Trials. 2022 May 18;23:416. doi: 10.1186/s13063-022-06364-z (PMC9116071; doi:10.1186/s13063-022-06364-z)
Supplement: Supplementary file 3 — Additional file 3. Statistical Analysis Plan. [file 13063_2022_6364_MOESM3_ESM.docx]

**Statistical Analysis Plan**

**PRIMA**

**Reducing the risk of *Plasmodium vivax* after *P. falciparum* infections in co-endemic areas - a randomized controlled trial**

**Version number: 1.0**

**Date: April 2022**

Contents

[1. Introduction 4](#_Toc98403072)

[2. Study design and objectives 4](#_Toc98403073)

[2.1. Summary of trial design 4](#_Toc98403074)

[2.2. Objectives 5](#_Toc98403075)

[Primary Objective 5](#_Toc98403076)

[Secondary Objectives 5](#_Toc98403077)

[2.3. Sample size 5](#_Toc98403078)

[3. Endpoints and Definitions 5](#_Toc98403079)

[3.1. Endpoints 5](#_Toc98403080)

[3.1.1. Primary efficacy Endpoints 5](#_Toc98403081)

[3.1.2. Secondary efficacy endpoints 5](#_Toc98403082)

[3.1.3. Safety Endpoints 6](#_Toc98403083)

[3.2. Definitions 6](#_Toc98403084)

[3.2.1. Symptomatic patients 6](#_Toc98403085)

[3.3. Handling of missing data and adjudication of Endpoints 6](#_Toc98403086)

[3.3.1. Handling of missing data on drug course 6](#_Toc98403087)

[3.3.2. Handling of missing data for Adverse Events 6](#_Toc98403088)

[3.3.3. Adjudication of day 28, 42 and 63 efficacy outcome assessment 6](#_Toc98403089)

[3.3.4. Adjudication for endpoint on parasite prevalence on day 0-3 7](#_Toc98403090)

[3.3.5. Adjudication for endpoint on fever clearance on day 0-3 7](#_Toc98403091)

[3.3.6. Adjudication for haemoglobin outcome 7](#_Toc98403092)

[3.4. Analyses Populations 8](#_Toc98403093)

[3.4.1. Efficacy population 8](#_Toc98403094)

[3.4.2. Safety population 8](#_Toc98403095)

[4. Analyses 8](#_Toc98403096)

[4.1. General analyses strategy 8](#_Toc98403097)

[4.2. Pooling individual patient data across sites 9](#_Toc98403098)

[4.3. Assessment of heterogeneity 9](#_Toc98403099)

[4.4. Demographic and baseline characteristics 9](#_Toc98403100)

[4.4.1. Demographic characteristics 9](#_Toc98403101)

[4.4.2. Disease characteristics at baseline 9](#_Toc98403102)

[4.4.3. Prior or concomitant medications 10](#_Toc98403103)

[4.5. Efficacy analyses 10](#_Toc98403104)

[4.5.1. Incidence risk of any *P. vivax* by day 63 10](#_Toc98403105)

[4.5.3. The incidence risk of any *P. vivax* parasitaemia at day 28 and day 42 10](#_Toc98403106)

[4.5.6. Proportion of patients with parasitaemia on day 1, 2 and 3 11](#_Toc98403107)

[4.5.7. Proportion of patients with fever on day 1, 2 and 3 11](#_Toc98403108)

[4.6. Safety analyses 11](#_Toc98403109)

[4.6.2. Proportion of patients vomiting any of their PQ doses during the 7-day supervised course 11](#_Toc98403110)

[4.6.3. Proportion of adverse events and serious adverse events 11](#_Toc98403111)

[4.6.4. The incidence risk of severe (Hb<5g/dl) and moderately severe anaemia (<7g/dl) and/or the risk for blood transfusion between day 3 and day 7 11](#_Toc98403112)

[4.6.5. The incidence risk of >= 25% fall in haemoglobin since baseline with and without hemoglobinuria at day 3 and day 7 12](#_Toc98403113)

[4.6.6. The incidence risk of >= 25% fall in haemoglobin to under 7g/dl with and without hemoglobinuria at day 3 and day 7 12](#_Toc98403114)

# Introduction

This Statistical Analysis Plan (SAP) provides a detailed and comprehensive description of the main pre-planned analyses for the clinical trial "Reducing the risk of *Plasmodium vivax* after *P. falciparum* infections in co-endemic areas - a randomized controlled trial”.

The main purpose of the trial is to assess the safety and efficacy of a 7-day course of high dose primaquine (PQ) treatment regimen in preventing recurrent *P. vivax* parasitaemia in G6PD normal patients following the treatment of uncomplicated *P. falciparum* malaria. The study conduct is described in Protocol HREC 19-3288 and registered with ClinicalTrials.gov under NCT03916003.

This document describes the statistical methods for the primary and secondary outcomes of the study as defined by the protocol.

# Study design and objectives

## Summary of trial design

This study is a multicentre, health care facility based, randomized, controlled, open label trial comparing high dose PQ treatment to prevent *P. vivax* parasitaemia in patients presenting with uncomplicated *P. falciparum* mono infection.

**Intervention**: Standard blood schizonticidal therapy plus 7 days of supervised PQ (7mg/kg total dose) administered once per day (1 mg/kg).

**Control arm**: Standard blood schizonticidal therapy with single dose of PQ (0.25mg/kg, if included in national treatment guidelines)

Patients tested initially and found to be G6PD deficient (<70% of adjusted male median value) will be excluded from the study.

PQ will be administered once per day with food (crackers or a biscuit) to reduce gastrointestinal side effects. All doses of study drugs will be supervised. If participants cannot visit the study centre, or fail to attend during the 7 days of supervised therapy, team members will visit them in their homes to encourage complete dosing. Treatment efficacy and patient safety will be ensured by close monitoring over a 63 day follow up period following a schedule of visits and corresponding clinical and laboratory examinations. Individuals will be reviewed regularly and sampled for haemoglobin and blood film examination.

## Objectives

### Primary Objective

To assess the safety and efficacy of a 7-day course of high dose PQ treatment regimen in preventing recurrent *P. vivax* parasitaemia by day 63 in G6PD normal patients following uncomplicated *P. falciparum* malaria.

### Secondary Objectives

- To assess the efficacy of a 7-day course of high dose PQ treatment regimen in preventing *P. falciparum* parasitaemia.
- To assess the efficacy of 7-day high dose PQ treatment in reducing gametocyte carriage of *P*. *falciparum*.
- To assess the safety and tolerability of high dose primaquine in patients with *P. falciparum*.

## 2.3. Sample size

This study was powered assuming that the risk of *P. vivax* after *P. falciparum* is highest after treatment with artemether-lumefantrine (AL), which is the schizonticidal treatment used in two of the three study sites (Bangladesh and Ethiopia). Assuming a risk of *P. vivax* after *P. falciparum* infection at day 63 after AL treatment of 41% and a reduction of this risk to 20% in the intervention arm, a total sample size of 322 patients will have 98% power at the two-sided 5% significance level. Assuming a loss to follow up rate of 20%, sample size will be increased to 403 across the sites in Ethiopia and Bangladesh where AL is used. We aim to recruit between 300 and 350 patients in Ethiopia, up to 50 in Bangladesh depending on the speed of recruitment.

We will aim to recruit 100 patients in Indonesia, which uses the schizonticidal treatment dihydroartemisinin-piperaquine (DHA-Pip). If we do not observe between site differences then estimates from the three sites will be combined giving >99% power for the primary outcome and additional power for the secondary outcomes.

# Endpoints and Definitions

## Endpoints

### Primary efficacy Endpoints

The incidence risk of any *P. vivax* parasitaemia at day 63

### Secondary efficacy endpoints

- The incidence risk of symptomatic *P. vivax* parasitaemia at day 63
- The incidence risk of any *P. vivax* parasitaemia at day 28 and day 42
- The incidence risk of any *P. falciparum* malaria at day 28, 42 and 63
- The incidence risk of *P. falciparum* gametocytaemia between day 7 and 63
- Parasite clearance on day 1, 2 and 3
- Fever clearance on day 1, 2 and 3

### Safety Endpoints

- The proportion of patients vomiting their medication on the day or enrolment within 1 hour of administration;
- The proportion of patients vomiting any of their PQ doses within 1 hour of administration;
- The proportion of adverse events and serious adverse events;
- The incidence risk of severe anaemia (Hb<5g/dl) and moderately severe anaemia (<7g/dl) and/or the risk for blood transfusion between day 3 and day 7;
- The incidence risk of ≥25% fall in haemoglobin since baseline with and without hemoglobinuria at day 3 and day 7;
- The incidence risk of ≥25% fall in haemoglobin to under 7g/dl with and without hemoglobinuria at day 3 and day 7.

## Definitions

### Symptomatic patients

“Symptomatic patients” are defined as patients with *P. vivax* parasitaemia and either a documented fever (axillary temperature ≥37.5°C) or a history of fever within the preceding 48 hours.

## Handling of missing data and adjudication of Endpoints

### Handling of missing data on drug course

Patients can have an incomplete course of treatment or data on drug administration may be missing. No imputation of treatment course will be made for patients with missing data.

### Handling of missing data for Adverse Events

For patients with missing data on adverse events the most conservative approach will be used:

| **Deviation** | **Action** |
| --- | --- |
| Start date of AE missing | Assume during study drug intake |
| End date of AE missing | No imputation |
| Date of start of study treatment administration missing | All AEs after randomization considered to have happened during study drug intake |
| Missing assessment of relationship to study treatment | Assume event to be possibly related |
| Missing severity assessment of AE | Assume highest severity |

### Adjudication of day 28, 42 and 63 efficacy outcome assessment

The clinical definition of treatment failure enables determination of the efficacy endpoint for schizontocidal activity. However, when the number of scheduled visits is incomplete the following rules will be applied^[[1]](#footnote-1)^:

| **Outcome assessment** | **Deviation** | **Action** |
| --- | --- | --- |
| **Day 28, 42 and 63** | More than 18 days without blood smear results | Lost to follow up on the day of last observation |
| **Day 28** | No blood smear results between day 25 and day 31 | Lost to follow up on day of last visit before day 25 |
| **Day 42** | No blood smear results between day 39 and day 45 | Lost to follow up on day of last visit before day 39 |
| **Day 63** | No blood smear results between day 60 and day 66 | Lost to follow up on day of last visit before day 60 |

### Adjudication for endpoint on parasite prevalence on day 0-3

For missed blood films (microscopy) the following rules will be applied^1^:

| **Parasitaemia by microscopy** | | | | **Action** |
| --- | --- | --- | --- | --- |
| **Day 0** | **Day 1** | **Day 2** | **Day 3** |  |
| Yes | Yes | No | Missing | Assumed no parasitaemia on day 3 |
| Yes | Yes | Missing | Yes | Assumed parasitaemia on day 2 |
| Yes | No | Missing | Missing | Assumed no parasitaemia on day 2 and 3 |
| Yes | Yes | Missing | No | Will not contribute to proportion of patients with parasitaemia on day 2 |
| Yes | No | Missing | No | Assumed no parasitaemia on day 2 |
| Yes | No | Missing | Yes | Will not contribute to proportion of day 2 with parasitaemia |
| Yes | Yes | Missing | Missing | Will not contribute to proportion of patients with parasitaemia on day 2 or 3 |

### Adjudication for endpoint on fever clearance on day 0-3

For missed temperature records, the same rules as for missing blood smear will apply.

### Adjudication for haemoglobin outcome

For missing haemoglobin measurements during follow up the following rules apply:

| **Outcome assessment** | **Deviation** | **Action** |
| --- | --- | --- |
| The incidence risk of severe anaemia (Hb<5g/dl) and moderately severe anaemia a(<7g/dl) and/or the risk for blood transfusion between day 3 and day 7 | Missing Hb measurement on day 3 or day 7 | Censored on last day of Hb measurement |
| The incidence risk of ≥ 25% fall in haemoglobin since baseline with and without hemoglobinuria at day 3 and day 7 | Missing Hb measurement on day 3 or day 7  Missing hemoglobinuria measurement | Censored on last day of Hb measurement  Censored on last day of hemoglobinuria measurement |
| The incidence risk of ≥25% fall in haemoglobin to under 7g/dl with and without hemoglobinuria at day 3 and day 7 | Missing Hb measurement on day 3 or day 7  Missing hemoglobinuria measurement | Censored on last day of Hb measurement  Censored on last day of hemoglobinuria measurement |

## Analyses Populations

### Efficacy population

For the efficacy analysis, both an intention-to-treat (ITT) and a per-protocol (PP) approach will be adopted. The ITT analysis will be the primary approach for comparison of the different drug treatments.

#### Intention to Treat

To provide a pragmatic comparison of the different drug treatments, the principle of intention-to-treat, will be the main strategy of analysis adopted for the primary and secondary endpoints. These analyses will be conducted on all patients assigned to the treatment groups as randomized, regardless of the study treatment received.

#### Per Protocol

An analysis based on a "per protocol" approach will be conducted following the causal framework presented in Hernan & Robins^[[2]](#footnote-2)^. The following participants will be excluded from the per protocol analyses:

- Ineligibility (either arising during the study or retrospectively having been overlooked at screening)
- Significant protocol deviation (e.g., given wrong total dose of primaquine)
- Incomplete treatment course

A causal diagram will be drawn to identify any pre- or post-randomisation confounders to guide the analysis approach.

### Safety population

For the analysis of safety outcomes, all patients who received any study drug are included in the safety analysis in the treatment group they actually received. This means also patients having vomited the first dose and having had their treatment discontinued will be included in this analysis.

# Analyses

## General analyses strategy

The primary endpoint analysis will use the combined data from all sites together, with adjustment for site effects. However, if there appears to be site differences between Bangladesh and Ethiopia compared to Indonesia (See sections 4.2 and 4.3 below) then the primary analysis will use data from Bangladesh and Ethiopia only. The analysis of secondary endpoints will include the third site in Indonesia.

## Pooling individual patient data across sites

As this is a randomized trial, with a common protocol and data collection and review tools across all sites, this pooled approach is expected to be valid. However, evidence for heterogeneity will be visually assessed and tested before deciding whether pooling of data across sites is justified. For the primary outcome, we will visually assess whether there are study differences across the three sites and when comparing Bangladesh and Ethiopia to Indonesia (see above).

## Assessment of heterogeneity

There is an expected degree of heterogeneity of exposure in different endemic settings, which might lead to heterogeneity between study sites regarding the treatment effect. Heterogeneity between study sites will be assessed visually using Forest plots. I-squared values (percentage of total variation across studies due to heterogeneity) will also be calculated.

If substantial levels of heterogeneity are detected, site specific analyses will be performed only. If no substantial levels of heterogeneity are detected, then pooled analyses will be performed.

## Demographic and baseline characteristics

Details of all patients screened, those who meet the study inclusion criteria, those who are eligible and randomized, those who are eligible but not randomized, those who withdraw from the study after randomization and those who are lost to follow-up will be summarized in a CONSORT flow diagram.

The number of patients discontinuing from the study will be tabulated by reason for study discontinuation. The number (%) of patients attending scheduled follow-up visits by study day will be reported.

The baseline value is defined as the last available value before randomization.

The following demographic and disease characteristics at baseline by treatment group will be presented.

### Demographic characteristics

• Gender: male / female

• Median Age (years), 25^th^-75^th^ percentiles, and range

• Age in classes: 12 months up to 5 years, 5 years up to 15 years, ≥15 years

- Median Weight (kg), 25^th^-75^th^ percentiles, and range

• Weight in classes: <9kg / 9-<18 kg/ 18 - <36 kg / ≥ 36 kg

### Disease characteristics at baseline

Specific disease history will include the parasite and gametocytes densities (/µL) at day 0, as well as axillary temperature (°C) (quantitative and if <37.5°C), and pulse rate (beats/minute), presented as median, 25th-75th percentiles, and range. The frequency and percent of symptoms (fever, chills malaise, fatigue, muscle pain, joint pain, headache, irritability, nausea, vomiting, diarrhea, abdominal pain, loss of appetite, jaundice, shortness of breath, dizziness, dark urine) at day 0 will be presented.

### Prior or concomitant medications

All medications taken within 28 days prior to randomization and the end of the study will be reported in the case report form.

All medications will be coded using the World Health Organization-Drug Dictionary (WHO-DD) or equivalent dictionaries.

- Prior medications are those the patient used prior (28 days before) to first study drug intake. Prior medications can be discontinued before first administration or can be ongoing during treatment phase.
- Concomitant medications are those used during the treatment with the study drug and during the study. Concomitant medications can be ongoing prior medication and/or continue into post treatment medication.

## Efficacy analyses

### Incidence risk of any *P. vivax* parasitaemia at day 63

The incidence risk (95% CI) of parasite recurrence within 63 days follow-up will be calculated using the Kaplan-Meier (KM) method for each trial arm as well as a comparison of the relative hazards between trial arms (Hazard Ratio (95% CI)) estimated from a Cox regression model for the time to the first recurrent episode with stratification for study site*.* The first episode of *P. vivax* (including mixed infections with *P. vivax*) will be treated as failure endpoint. Patients presenting with parasitaemia other than *P. vivax* will be censored at the day of occurrence.

Comparisons will be made between the intervention and control arm. The analyses will be performed in the ITT and the PP population with stratification for site.

- - 1. **The incidence risk of symptomatic *P. vivax* parasitaemia at day 63**

This analysis will be performed as described in section 4.5.1. The first symptomatic episode of *P. vivax* will be treated as failure endpoint. Patients presenting with symptomatic parasitaemia other than *P. vivax* will be censored at the day or occurrence. Patients with asymptomatic parasitaemia will be censored at the time of occurrence.

Comparisons will be made between the intervention and control arm. The analyses will be performed in the ITT and the PP population for, controlling for site.

### The incidence risk of any *P. vivax* parasitaemia at day 28 and day 42

This analysis will be performed as described in section 4.5.1. with endpoints on day 28 and 42. Comparisons will be made between the intervention and control arm. The analyses will be performed in the ITT and the PP population controlling for site.

- - 1. **The incidence risk of any *P. falciparum* malaria at day 28, 42 and 63**

These analyses will be performed as described in section 4.5.1. The first episode of *P. falciparum* will be treated as failure endpoint. Patients presenting with parasitaemia other than falciparum will be censored on the day of occurrence.

Comparisons will be made between the intervention and control arm. The analyses will be performed in the ITT and the PP population controlling for site.

- - 1. **The incidence risk of *P. falciparum* gametocytaemia between day 7 and 63**

This analysis will be performed as described in section 4.5.1. The first episode of *P. falciparum* gametocytemia after day 7 will be treated as failure endpoint. Patients presenting with parasitaemia other than falciparum or without sexual parasites will be censored on day of occurrence.

Comparisons will be made between the intervention and control arm. The analyses will be performed in the ITT and the PP population controlling for site.

### Proportion of patients with parasitaemia on day 1, 2 and 3

*P. falciparum* parasite clearance will be presented using the proportions of patients who remain parasitaemic on each day (days 1 to 3).

### Proportion of patients with fever on day 1, 2 and 3

Fever clearance time will be presented as the proportion of patients who were febrile on each day (days 1 to 3). This analysis will be restricted to patients who were febrile (<37.5°C) at enrolment.

## Safety analyses

The primary safety concerns regarding primaquine use pertain to the risk of haemolysis and gastroenterological adverse events.

#### Proportion of patients vomiting their medication within 1 hour of administration

Proportion of patients with vomiting within 1 hour of administration of a dose will be calculated for each arm. The absolute difference between the intervention arm and the control arm will be presented.

### Proportion of patients vomiting any of their PQ doses during the 7-day supervised course

Proportion of patients with vomiting during the first 7 days of treatment will be calculated.

### Proportion of adverse events and serious adverse events

The proportion of patients with one or more adverse events and serious adverse events within the first 7 days and until day 42 will be presented. Events will be divided into related and unrelated events with respect to the treatment. The absolute difference between treatment groups in the proportion of patients (two-sided 95% CI) with AEs and SAEs will be calculated for those time points.

### The incidence risk of severe (Hb<5g/dl) and moderately severe anaemia (<7g/dl) and/or the risk for blood transfusion between day 3 and day 7

The incidence risk will be calculated using the Kaplan-Meier method. Patients who develop severe anaemia (defined as i) dropping <7g/dl; ii) dropping <5g/dl and/or iii) requiring transfusion) will be considered failures. Patients lost to follow up or with missing measurements will be censored at the time of the last visit.

Comparisons will be performed between the intervention and the control arm by comparing the relative hazards (Hazard Ratio (95% CI)) estimated from a Cox regression model with stratification for study site and adjustment for baseline haemoglobin.

### The incidence risk of >= 25% fall in haemoglobin since baseline with and without hemoglobinuria at day 3 and day 7

The incidence risk will be calculated using the Kaplan-Meier method. Patients who develop a drop in Hb >= 25% compared to baseline will be considered failures. Patients lost to follow up or with missing measurements will be censored at the time of the last visit.

Comparisons will be performed between the intervention and the control arm by comparing the relative hazards (Hazard Ratio (95% CI)) estimated from a Cox regression analysis with stratification for study site and adjustment for baseline haemoglobin.

### The incidence risk of >= 25% fall in haemoglobin to under 7g/dl with and without hemoglobinuria at day 3 and day 7

The incidence risk will be calculated using the Kaplan-Meier method. Patients who develop a drop in Hb >=25% compared to baseline and drop <7g/dl will be considered failures. Patients lost to follow up or with missing measurements will be censored at the time of the last visit.

Comparisons will be performed between the intervention and the control arm by comparing the relative hazards (Hazard Ratio (95% CI)) estimated from a Cox regression analysis with stratification for study site and adjustment for baseline haemoglobin.

1. WWARN. Clinical Module, Data Management and Statistical Analyses Plan. 2013; Available from: http://www.wwarn.org/sites/default/files/ClinicalDMSAP.pdf [↑](#footnote-ref-1)
2. Hernan & Robins. Per-protocol Analyses of Pragmatic Trials. N Engl J Med. 2017 Oct 5;377(14):1391-1398. doi: 10.1056/NEJMsm1605385. [↑](#footnote-ref-2)
